# Role of the ortho-bridge system in the length unstable subtrochanteric femur fracture in school going children: a retrospective clinical study of 19 cases

**DOI:** 10.3389/fped.2023.1306076

**Published:** 2023-11-22

**Authors:** Mingjing Li, Jian Xu, Jiang Xiang, Chunquan Zhu, Zonghui Dai, Fan Li

**Affiliations:** Department of Pediatric Orthopedics, Wuhan Fourth Hospital, Wuhan, China

**Keywords:** subtrochanteric femur fracture, ortho-bridge system, children, length unstable, retrospective study

## Abstract

**Background:**

Treating subtrochanteric femur fractures in pediatric patients remains challenging, and an optimal fixation device has yet to be established. This study aimed to asess the clinical and radiological outcomes of Ortho-Bridge System (OBS) treatment for length-unstable subtrochanteric femur fractures in children aged 5–16 years.

**Methods:**

We conducted a retrospective review of pediatric patients with subtrochanteric femur fractures treated with OBS between January 2018 and December 2021. The study included 19 children (12 boys, 7 girls) with an average age of 10.4 ± 2.6 years. Two of the patients had pathological fractures associated with aneurysmal bone cyst. Demographic information, mechanism of accident, fracture type, associated neurovascular injuries, surgical duration and blood loss, were collected from the hospital database. Time to union and postoperative complications were recorded. Clinical and radiological outcomes were assessed using the Harris scoring system at the latest follow-up.

**Results:**

Injuries resulted from vehicle accidents in 10 patients (52.6%), falls over 3 meters in height in 3 patients (15.8%), and sports-related injuries in 6 patients (31.6%). The average patient weight was 41.5 kg (range: 21–78). Of the fractures, 14 (73.7%) were complex, and 5 (26.3%) were spiral. The average surgical duration was 111 min (range: 90–180), and the average surgical blood loss was 134 ml (range: 70–300). The mean time to union was 12.7 weeks (range: 8–16). No cases of infection, malunion, implant failure, or femoral head osteonecrosis were reported. Leg length discrepancy of 10 mm was observed in one patient. All patients achieved excellent results according to the Harris scoring system.

**Conclusion:**

This study suggests that the OBS may serve as an effective alternative fixation option for managing length-unstable subtrochanteric femur fractures in school-aged children.

## Introduction

Subtrochanteric femur fractures are uncommon in children, accounting for only 4%–10% of all pediatric femoral fractures (1, 2). Despite their low frequency, these fractures require emergency care and have a high hospitalization rate. According to Pombo and Shilt's definition, subtrochanteric fractures are those occurring within 10% of the total femur length below the lesser trochanter (3). These fractures are unstable due to the strong muscular forces on the proximal fragment, causing flexion, abduction, and external rotation. Treatment options depend on the patient's age, weight, fracture pattern, associated injuries, and the surgeon's experience (4, 5). While spica casting remains a common treatment for patients under 5 years old, older children (aged 5–16 years) typically require surgery (6).

Various methods are available for managing femoral fractures in children, such as titanium elastic nails (TENs), locking plates, or external fixators (6). The optimal treatment for subtrochanteric femur fractures in school going children is still controversial. TENs are a well-established surgical option for children under 11 years of age, whereas rigid locking intramedullary nails are recommended for adolescent patients (7). With advancements in surgical techniques, TENs have been extended to include length-stable (transverse or short oblique) fractures in the proximal 1/3 of the femoral diaphysis and subtrochanteric fractures (8). However, in cases of spiral or comminuted subtrochanteric fractures, or fractures in heavy children (over 45 kg), TENs have a high failure rate. In such situations, locking plates are a viable alternative. Pediatric proximal femur locking plates may not always be readily available in developing countries, and standard locking plates used for subtrochanteric femur fractures may not provide sufficient fixation points in the short proximal femur segment, potentially compromising stability.

**Figure 1:**
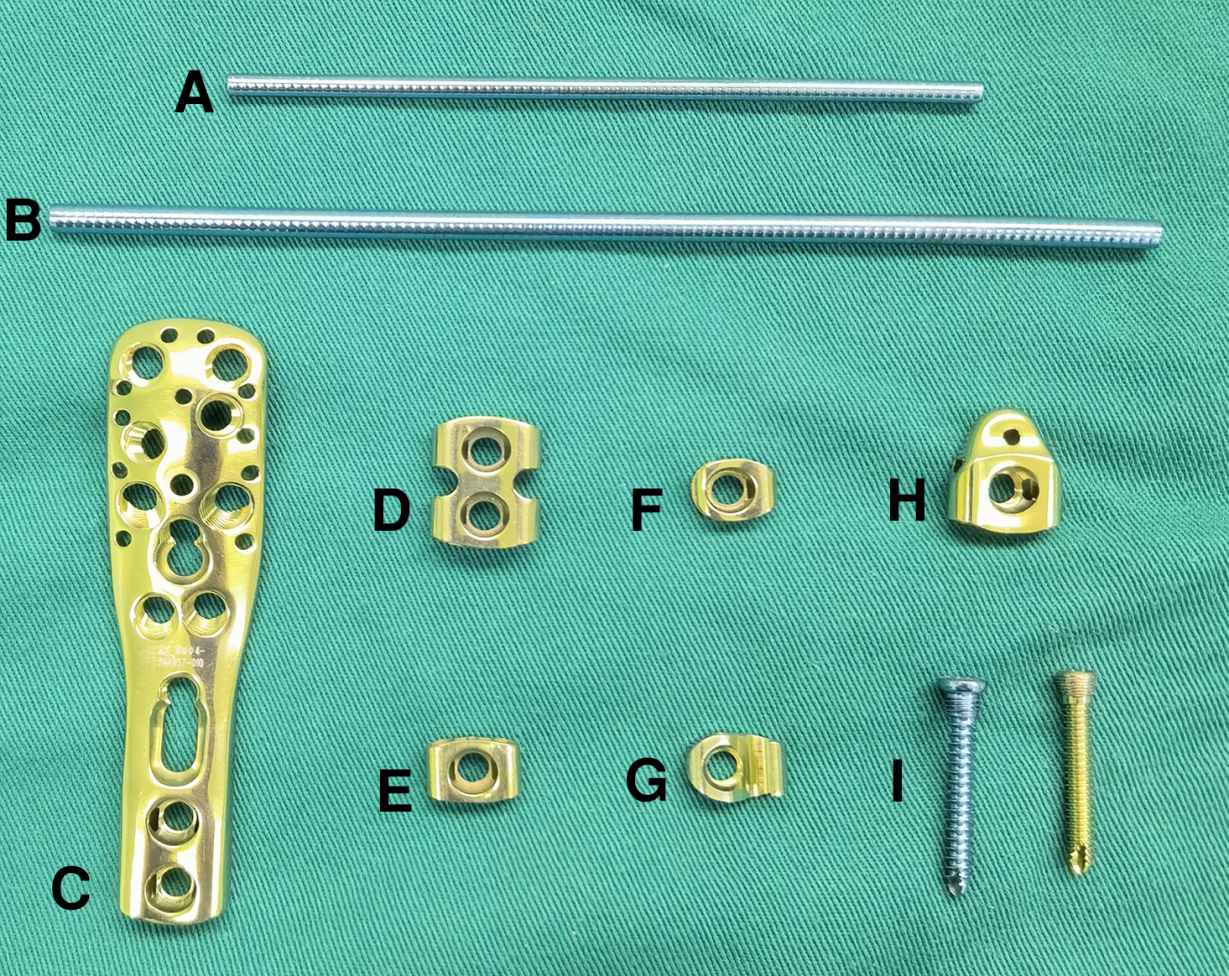
Basic components of the ortho-bridge system. (**A,B**) Connecting rods with different diameter, (**C–H**) various clamps, (**I**) screws.

**Figure 2:**
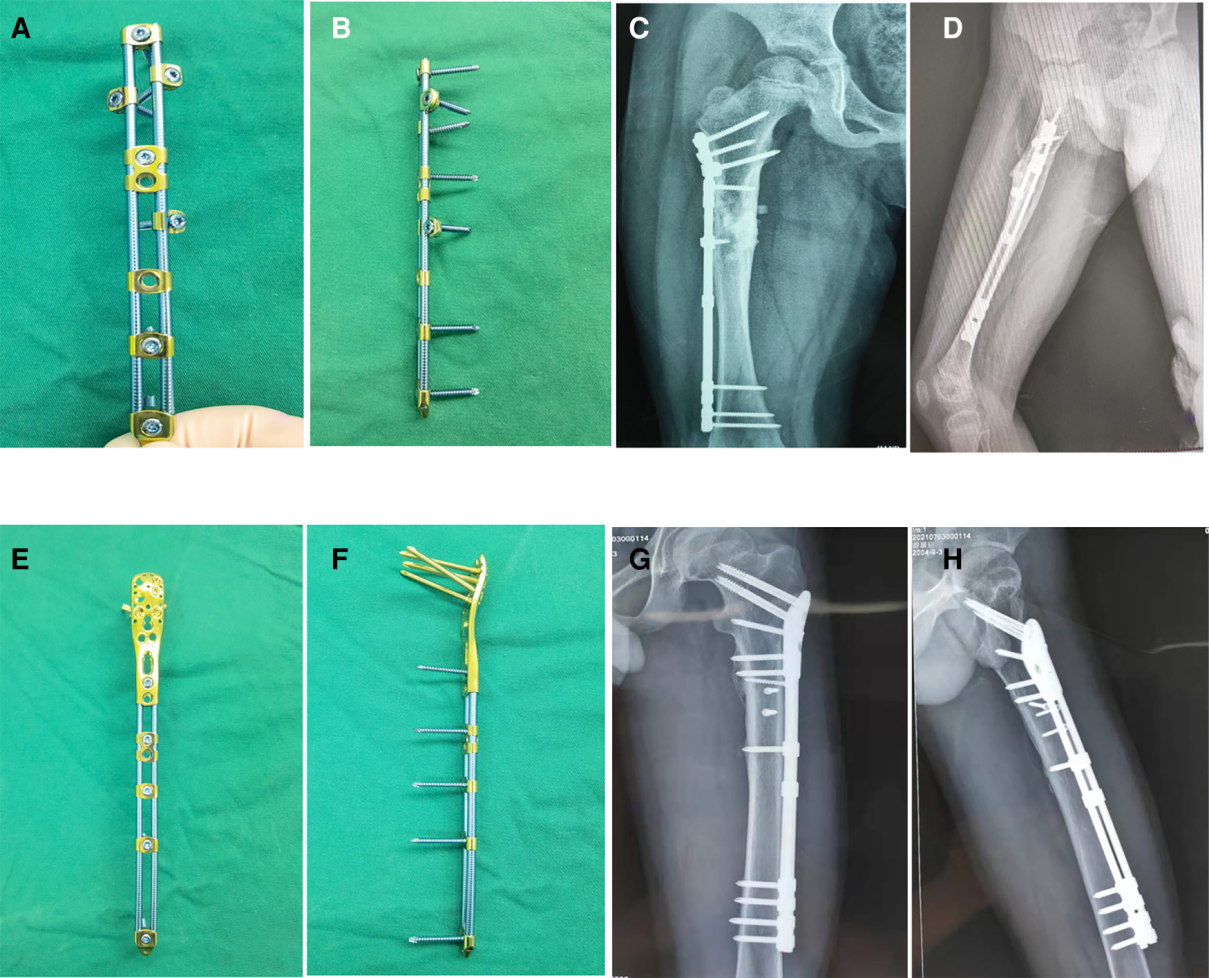
Models and typical cases of OBS fixation. (**A,B**) Model of a double-rod OBS. (**C,D**) OBS fixation for subtrochanteric femur fracture of a 6-year-old girl. **(E,F**) Model of the OBS composed of double rods and special anatomical clamp. (**G,H**) OBS fixation for subtrochanteric femur fractures of a 15-year-old boy.

In this article, we introduce another internal fixation instrument for subtrochanteric fractures, namely the Ortho-Bridge System (OBS). The OBS was developed and designed in China with independent intellectual property rights held by Walkman Biomaterial Co., Ltd., Tianjin, China (patent number: ZL200510010654.3). It primarily comprises rods, clamps, and screws (Figure 1, Supplementary file S1), which can be freely combined by the surgeon to form individual internal fixation complexes. Unlike the single position and direction of screws in standard plate fixation, OBS screws offer adjustable positions and directions to match specific situations, enhancing versatility and ease of use (Figure 2). Angular screw options provide better resistance to pull-out and improved stability. This three-dimensional adjustability of OBS screws stands as the primary advantage of this system over traditional plate fixation, making it a preferred choice for treating pediatric subtrochanteric fractures. Biomechanical and clinical analyses have demonstrated favorable results with OBS for pelvis and long bone metaphyseal fractures, particularly in aged patients with osteoporosis (9). To the best of our knowledge, there have been limited reports on the use of OBS in the treatment of pediatric subtrochanteric femur fractures. Therefore, this retrospective study was conducted to assess the effectiveness of OBS in managing length-unstable subtrochanteric femur fractures in school going children.

### Patients and methods

The charts and radiographs of all pediatric patients with subtrochanteric femur fractures ﬁxed with OBS (Walkman Biomaterial Co., Ltd., Tianjin, China) at our institution between January 2018 and December 2021 were reviewed retrospectively. A subtrochanteric femur fracture was deﬁned as a fracture occuring within 10% of the total femur length below the lesser trochanter. Fracture instability was characterized as “length unstable” if it was complex with multiple fragments, long oblique or spiral where the length of the fracture was twice as long as the femur's diameter at the fracture level (10). Inclusion criteria included: (1) patients aged 5–16 years with open growth plates at the time of fracture; (2) length-unstable subtrochanteric femur fractures treated with OBS; and (3) a follow-up period exceeding 12 months. Exclusion criteria included: (1) patients aged over 16 years or those with closed growth plates at the time of fracture; (2) length-stable (transverse or short oblique) femoral fractures; (3) fractures associated with neurovascular injuries; (4) open fractures; (5) cases of refracture or nonunion following prior surgery; and (6) a follow-up period of less than 12 months. The study protocol was approved by the Ethics Committee of Wuhan Fourth Hospital (No: KY2023-034-01).

Demographic information, mechanism of accident, fracture types, and associated neurovascular injuries were collected from the hospital database. Operation time, blood loss, duration of follow-up, time to union and postoperative complications were also documented. Anteroposterior (AP) and lateral radiographs of the femur were assessed for union, implant migration, alignment, or re-fracture in the case of recent trauma. Radiographic union was deﬁned as the bridging of three out of the four cortices and the disappearance of the fracture line on radiographs. Secondary displacement referred to a loss of bone alignment accompanied by >10° of angulation, rotation or impaired bone length. Malunion was defined as varus or valgus >10° or anterior bowing >15°. All the patients were clinically followed until the OBS was removed and they regained normal function. In our institution, hardware removal was routinely performed at 6–12 months after radiographic fracture union. Clinical assessments included evaluating limb lengths, alignment, and hip motion. Hip function was assessed using the Harris Hip Score scale at the latest follow-up (11).

### Surgical technique

A brief description of the technique is given here. The surgeries were conducted by three experienced pediatric orthopedists. All patients were positioned supine and given general anesthesia. A straight lateral incision (several centimeters long) was initiated at vastus ridge level on the greater trochanter. The fascia lata was opened, and a periosteal elevator was used to gently lift the vastus lateralis muscle from the bone, without causing harm to the periosteum. After exposing the bone fragments, the fracture was reduced under direct vision with traction manually, or via a fracture table. Following the indirect reduction of the fracture, a submuscular route was prepared for plate insertion along the femur's shaft using an elevator. For more minimally invasive plate placement, the entry point was typically located laterally to the distal femoral metaphysis, where a 3–5 cm incision was made anterolaterally.

The Ortho-Bridge System (OBS) consists of rods and clamps of various sizes that can be freely matched according to patient's age, weight, and fracture patterns. A common combination involves a double-rod system combined with a proximal femoral or humeral anatomical clamp. Rods with diameters of 3.5 mm or 4.5 mm are respectively paired with screws of corresponding sizes and modules. The connecting rods can be bent and shaped. Three to four points of fixation on the main proximal and distal fragments allow for stability. In order to disperse the stress, the rods length is maintained at a minimum of four to five times the femur's diameter. The distal end of the OBS can be positioned laterally to the distal femoral metaphysis, with screws introduced through small lateral incisions using a minimally invasive technique.

**Figure 3:**
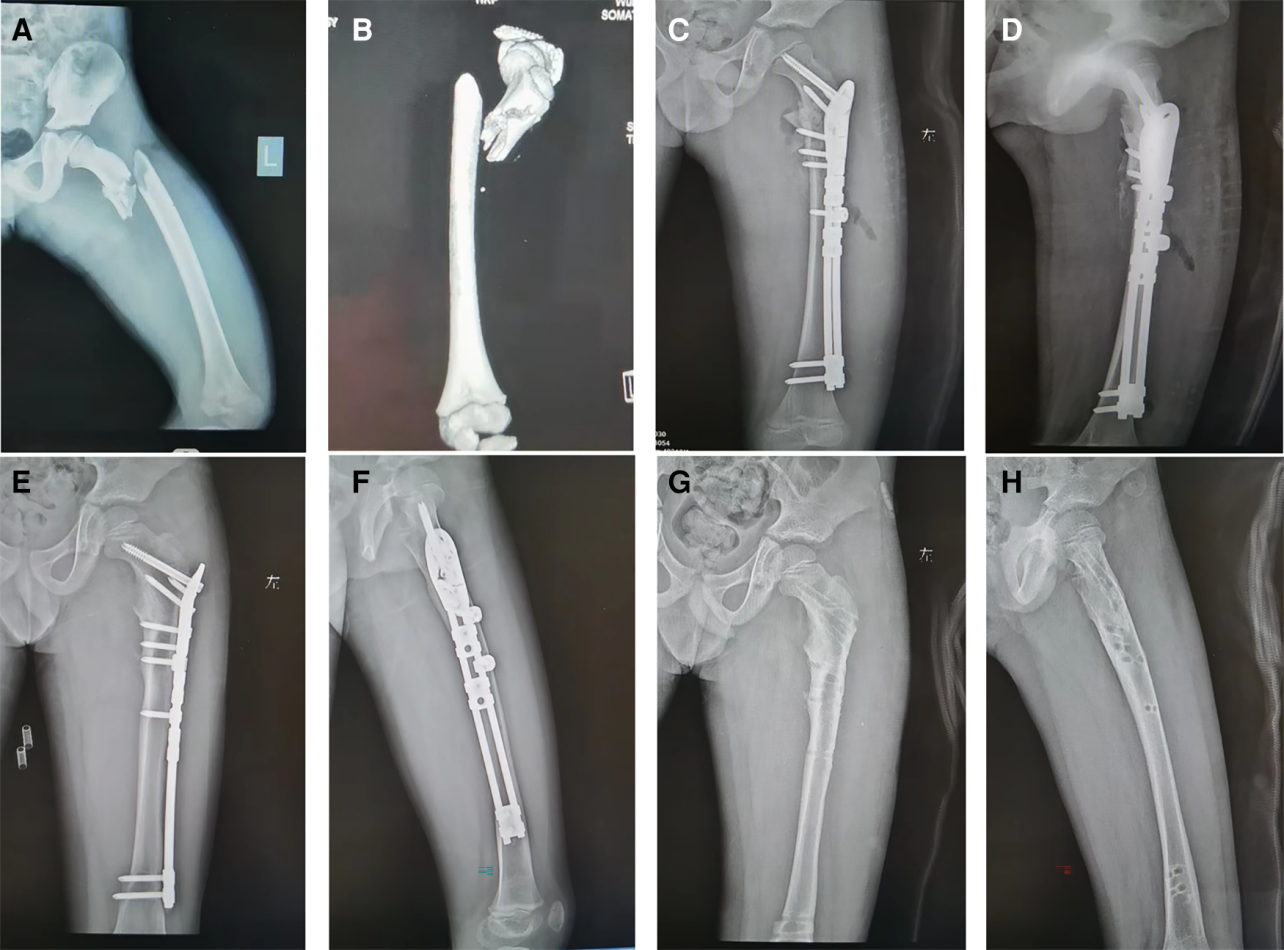
OBS fixation for a typical length-unstable subtrochanteric femur fracture. Preoperative (**A,B**) and early postoperative (**C,D**) radiographs of a 7-year-old boy. The fracture healed at 12 weeks follow-up visit (**E,F**), and the implant was removed after one year (**G,H**).

**Figure 4:**
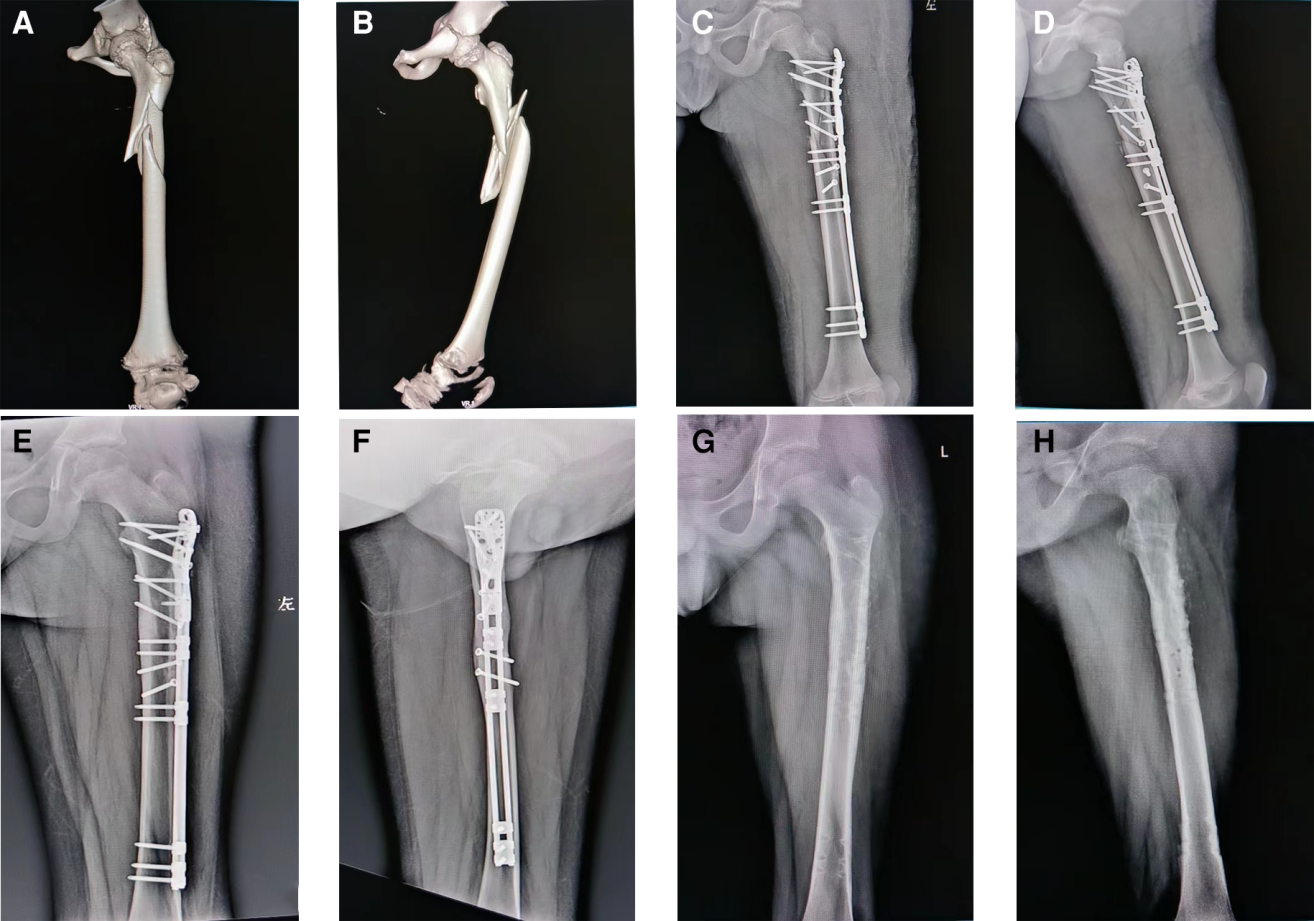
OBS fixation for a complex subtrochanteric femur fracture. Preoperative (**A,B**) and early postoperative (**C,D**) radiographs of a 10-year-old boy. The fracture healed at 16 weeks follow-up visit (**E,F**), and the implant was removed after one year (**G,H**).

Upon selecting two rods of appropriate length, three or four sliding clamps were affixed to the connecting rods. A proximal femoral or humeral anatomical clamp was placed on the lateral aspect of the proximal femur to match its contour. The push-pull technique with an articulated tension device or spreader and the reduction forceps is an elegant and regularly used way to correct residual deformity. After ﬁxation of the OBS to main fragments with 2–3 screws, a tension device was applied between the clamps to either distract the fracture or achieve interfragmentary compression, facilitating complete proximal reduction. The distal screws have to be slightly loosened to enable the clamps to slide on the rods, before being retightened. Multiple locking screws at various angles and long screws extending up to the femoral neck were utilized for proximal ﬁxation (Figures 3, 4). The set screws were inserted into the clamps to tighten the connection rods and clamps.

In cases of complex fragmental fractures, we can adapt by using lateral sliding clamps on the rods and inserting screws at different angles to fix the fragments. The small fragment is gently reduced with a small hook. Three-dimensional screws placement can increase the stability of fixation. Fluoroscopy should be taken to make sure femoral alignment was reduction and the screws do not violate the growth plate. At last, routine closure is performed for the surgical wound.

To alleviate postoperative pain and maintain correction, all patients are immobilized in a long leg lateral splint for duration of 3–4 weeks after surgery. After removing the splint, knee and hip movement was started. Patients are advised to refrain from weight-bearing activities until radiographic healing of the fractures was confirmed.

### Statistical analysis

Quantitative data was expressed as the mean and range. Statistical analysis was performed using the IBM SPSS statistics version 18 software (SPSS Inc., Chicago, Illinois).

## Results

As shown in Table 1, there were 12 boys and 7 girls with an average age at the time of surgery of 10.4 years (range: 6.2–15.1). The causes of the injuries were as follows: vehicle accidents in 10 patients (52.6%), falls over 3 meters in height in 3 patients (15.8%), and sport-related injuries in 6 patients (31.6%). The average weight of the patients was 41.5 kg (range: 21–78). The affected side was the right femur in 6 cases (31.6%) and the left in 13 cases (68.4%). Two patients sustained pathologic fractures involving aneurysmal bone cyst (ABC). One patient sustained an ipsilateral tibia fracture, and another had an ipsilateral radius fracture. All fractures were closed fractures without any neurovascular injuries. The study included 14 (73.7%) complex fractures and 5 (26.3%) spiral fractures. The average time from injury to surgery was 4.5 days (range: 3–8).

**Table 1:** Patient demographics, fracture data, operative data, complications, and functional outcomes.

Cases	Gender	Age (year + month)	Weight (Kg)	Mechanism of injury	Pathology	Associated injury	Type of fracture	Operation time (min)	Blood loss (ml)	Time to union (weeks)	Complications	Harris score
1	M	8 + 7	30	Sports injury			S	90	80	9		Exc
2	F	6 + 5	21	Sports injury	ABC		C	110	150	16		Exc
3	M	13 + 2	70	Vehicle accident			C	120	180	12		Exc
4	M	15 + 1	78	Vehicle accident			S	90	90	12		Exc
5	M	13 + 8	60	Vehicle accident			C	120	150	12		Exc
6	M	14 + 3	60	Vehicle accident			C	110	130	12		Exc
7	M	10 + 3	41	Vehicle accident		Ipsilateral radius fracture	C	180	300	16		Exc
8	M	9 + 7	32	Fall from height			C	110	120	12		Exc
9	F	7 + 4	28	Vehicle accident			C	100	100	12		Exc
10	F	11 + 2	40	Sports injury			C	120	130	14		Exc
11	F	10 + 6	38	Vehicle accident			C	120	140	12		Exc
12	M	8 + 5	30	Sports injury	ABC		C	150	250	16		Exc
13	M	11 + 8	40	Vehicle accident			C	110	110	16		Exc
14	M	6 + 2	25	Fall from height			S	90	70	8		Exc
15	M	12 + 5	63	Vehicle accident		Ipsilateral tibia fracture	S	140	170	16	LLD of 1 cm	Exc
16	M	8 + 9	29	Sports injury			S	70	70	10		Exc
17	F	11 + 2	38	Sports injury			C	90	100	13		Exc
18	F	7 + 11	30	Vehicle accident			C	90	100	12		Exc
19	F	10 + 5	35	Fall from height			C	100	120	12		Exc

M, male; F, female; ABC, aneurismal bone cyst; S, spiral; C, complex; LLD, leg length discrepancy; Exc, excellent.

The average operation time was 111 min (range: 90–180) and the average surgical blood loss was 134 ml (range: 70–300). The average hospitalization time was 11.5 days (range: 7–14). The mean follow-up period was 14.5 months (range: 12–18). Radiographic follow-up revealed that the mean time to achieve union was 12.7 weeks (range: 8–16). Throughout the follow-up period, there were no patients with superﬁcial or deep infection, implant failure, malalignment, or refracture. Additionally, no cases of osteonecrosis of the femoral head or heterotopic bone formation were observed. At the latest follow-up, one case exhibited limb lengthening of 10 mm on the operative side, attributable to excessive traction during fracture fixation. All patients had their implants removed after fracture union, with no complications reported. All patients achieved excellent results based on the Harris scoring criteria at the latest follow-up.

## Discussion

Pediatric subtrochanteric femur fractures can be challenging to treat due to the instability and displacement of the short metaphyseal fragments, as well as limited remodeling potential of the proximal femur. So far, there are several methods of managing subtrochanteric femur fractures, including simple spica casting for younger children and surgeries with internal or external ﬁxation for older children. Spica casting following traction requires a prolonged hospital stay and demonstrates the limited capability of restoring the limb length and alignment (6). The most suitable ﬁxation device for treating pediatric subtrochanteric femur fractures has not been determined. In our current study, we have demonstrated that using OBS for ﬁxing these fractures can lead to good functional outcomes.

As shown in Table 2, there are several ﬁxation device options for subtrochanteric femur fractures, including TENs, standard locking plates, and external ﬁxators (12–15). TENs has been widely applied in treating patients with length stable fractures and those weighing less than 49Kg. However, high failure rate were reported when it used in managing length unstable, proximal or distal femur fractures, and in heavy children. Sink et al. reported a 22% complication rate in fractures of the proximal 1/3 of the femur treated with TENs (18). To enhance the strength of elastic nails, many scholars have made improvements in the placement technique and the number of nails used. It has been noted that pushing the nail into the greater trochanter apophysis or the posterior femoral neck cortex has improved the mail's support strength (8). Satisfactory results have been achieved in the treatment of length stable fractures, such as transverse or short oblique subtrochanteric fractures. Regarding long oblique subtrochanteric fractures, a comparative study by Tang et al. found similar outcomes between TENs and plate fixation (16). However, their study did not include complex fractures, and the age range of the patients was between 7 and 10 years. In some young children, the narrow medulla of the femur cannot accommodate three TENs, and this method does not improve stability. In our study, out of the 19 cases, 14 had complex fractures, making it challenging to achieve local three-point support. The short proximal segment tends to flex and rotate due to the pull of attached muscles. Complications after TENs fixation are related to the severity of comminuted fractures, and the probability of complications is significantly higher in the group with unstable fractures.

**Table 2:** Comparision of our study with those in the literature reporting results of treatment for pediatric subtrochanter femur fractures.

Study	Number of cases	Age (year)	Weight (Kg)	Femur fracture location	Fracture type	Treatment method	Time to union	Major complications
Abdelgawad et al. (12)	58	3.6–15.7	12–71	All types	All types	SP	Average 13.3 weeks	One had implant failure,one had deep infection,10 had an average LLD of 9.9 mm
Parikh et al. (13)	36	5–8.8	18–29	ST	All types	TEN	NA	Two had malunion >1°, one had fracture at nail insertion site,one had loss of reduction and nail repositioned,one had LLD of 2 cm
Galal et al. (14)	14	3.8 11.5	NA	ST	All types	EF	6–12 weeks	Four had pin site infection
Cha et al. (8)	17	5–12	20–38	ST	All types	TEN	3–6 months	Three had malunion >5, five had LLD of 1–2 cm
Lakhani et al. (15)	30	8–14	NA	ST	Spiral or complex	SP	8–18 weeks	One had LLD of 3 mm
Basa et al. (5)	20	4.6–14.4	NA	ST	All types except severely comminuted	TEN	4–7 months	Three had malunion <5°
Hong et al. (16)	16 (TEN) 16 (plate)	8.4 ± 1.5 8.4 ± 1.4	35.8 ± 1.0 36.2 ± 1.3	ST	All types except severely comminuted	TEN LP	11.8 ± 2.0 weeks 13.2 ± 1.7 weeks	None
Danişman et al. (17)	9	7–12	NA	ST	All types	LP	6–10 weeks	One had LLD of 10 mm,one had coxa valga and LLD of 16 mm
Current study	19	6.2–15.1	21–78	ST	Complex or spiral	OBS	8–16 weeks	One had LLD of 10 mm

TEN, titanium elastic nails; NA, not available; ST, subtrochanteric; SP, submuscular plate; LP, locking plate; EF, external fixator; LLD, Leg length discrepancy; OBS, Ortho-bridge system.

There are 5 children in the article, weighing over 50 kg. Multiple studies have indicated that children weighing more than 49 kg are five times more likely to experience complications after undergoing elastic nail surgery compared to other children (10). Compared to TENs, rigid locking intramedullary nails have the advantages such as strong stiffness, anti torsion force, and biomechanical stability. However, rigid nailing requires an entry point located laterally to the tip of the trochanteric physis to prevent harm to blood vessels near the proximal femur. It is suitable only for older patients who have a sufficiently large medullary canal for nail placement. There is a potential risk of growth arrest in the greater trochanter apophysis, resulting in coxa valga and heterotopic bone formation when the trochanteric physis is violated (19). Very proximal and distal femoral fractures remain challenging to treat with rigid nails. In case of polytrauma or open injuries, external ﬁxation is considered an alternative (14). However, it has limited applicability in closed fractures due to an increased risk of pin tract infection, refracture, and loss of reduction. Additionally, in subtrochanteric femur fractures, there may not be enough space for pin placement in the proximal femoral segment.

For decades, plate fixation has been used for length unstable femur fractures, and has delivered anatomical reduction and adorable clinical outcomes (20). Patrikov et al. have used reconstruction or bridging steel plates to effectively treat comminuted proximal femoral fractures in children (21). However, the use of standard locking plates for these pediatric subtrochanteric femur fractures, as shown in Figures 3, 4, may offer only minimal points of fixation due to the short proximal segment, which might not provide a robust construct. Danişman et al. have achieved good results in treating pediatric subtrochanteric fractures by using adult proximal humerus locking plates (17). This can be a viable alternative, especially in regions where pediatric proximal femur locking plates are not available.

In our institution, we have adopted the OBS for treating length-unstable subtrochanteric fractures, and this approach has yielded satisfactory clinical outcomes. The OBS is a novel type of fracture fixation device, which combines the structural principles of locking plates, external fixators, and spine screw-rod systems. The system comprises connecting rods, fixing clamps, and screws. During the operation, the choice and assembly of fixed and anatomical clamps, as well as connecting rods, can be customized according to the patient’s age, weight, and fracture type to achieve personalized fixation. Indirect reduction procedures and less invasive operative techniques are preferred because they cause minimal damage to the blood supply to the fragments, contributing positively to the healing process. The anatomical module of the proximal humerus or femur allows the placement of multiple locking screws into the patient’s femoral neck at different angles, resulting in increased angular stability. For younger children, when the size of anatomical clamps is too large, as shown in Figure 2, the proximal end of the connected rods can be pre-bent, and a nail can be inserted into the femoral neck through a single-hole clamp, or slide clamps can be attached to the rod, allowing screws to be placed in various directions to achieve three-dimensional fixation within limited space.

Biomechanical analysis indicates that the OBS can withstand a higher axial load and distribute the load to the femur, making it better suited for early functional exercise and fracture healing (22). The combination of connecting rods and clamps can better disperse stress to avoid stress concentration and reduce the risk of metal fracture. When bearing weight, the connecting rods can slide slightly in the axial direction to form dynamic compression which stimulates callus formation, prevents stress shielding, and reduces the risk of osteoporosis. In our study, all fractures healed well, with an average time to union of 12 weeks, including pathological fractures. We achieved favorable outcomes using the OBS for treating length-unstable subtrochanteric femur fractures in children aged 5–16 years. Although one child had a slight leg length discrepancy, it did not affect limb function. However, the OBS has certain disadvantages when compared to intramedullary devices. These include traditional plate-related issues like blood loss due to open reduction, extensive soft tissue dissection, and longer incision scars.

Our study has several limitations. First, this retrospective study involved a small number of cases, giving the low incidence of pediatric subtrochanteric femur fractures. Second, there was no control group with different implants. Third, the OBS is relatively costly compared to TENs or classic compression plates, which is a significant drawback to consider in fracture management. To further assess the technique's effectiveness, well-designed, multicenter and prospective trials comparing it with pediatric proximal femur locking plating or intramedullary nailing are needed. We will try to gather more data and enhance the study's evidence level in the future work.

## Conclusion

This study reports the promising outcomes of using OBS for ﬁxing subtrochanteric femur fractures with length-unstable patterns in children of 5–16 years old. The OBS offers customization based on the patient's age, weight, and fracture type, allowing for individualized treatment. The use of screws in various directions enhances fixation strength. In summary, this system offers personalization, flexibility, convenience, and stability, making it a viable alternative for treating pediatric subtrochanteric femur fractures.

## Data Availability

The original contributions presented in the study are included in the article/**Supplementary Materials**, further inquiries can be directed to the corresponding author.

## Supplementary material

Supplementary File S1 The Ortho-Bridge System introduction which come from the official website of Walkman Biomaterial Co., Ltd.
