# Supplementary material for: Role of the ortho-bridge system in the length unstable subtrochanteric femur fracture in school going children: a retrospective clinical study of 19 cases
Source: Front Pediatr. 2023 Nov 22;11:1306076. doi: 10.3389/fped.2023.1306076 (PMC10703289; doi:10.3389/fped.2023.1306076)

LEPU MEDICAL

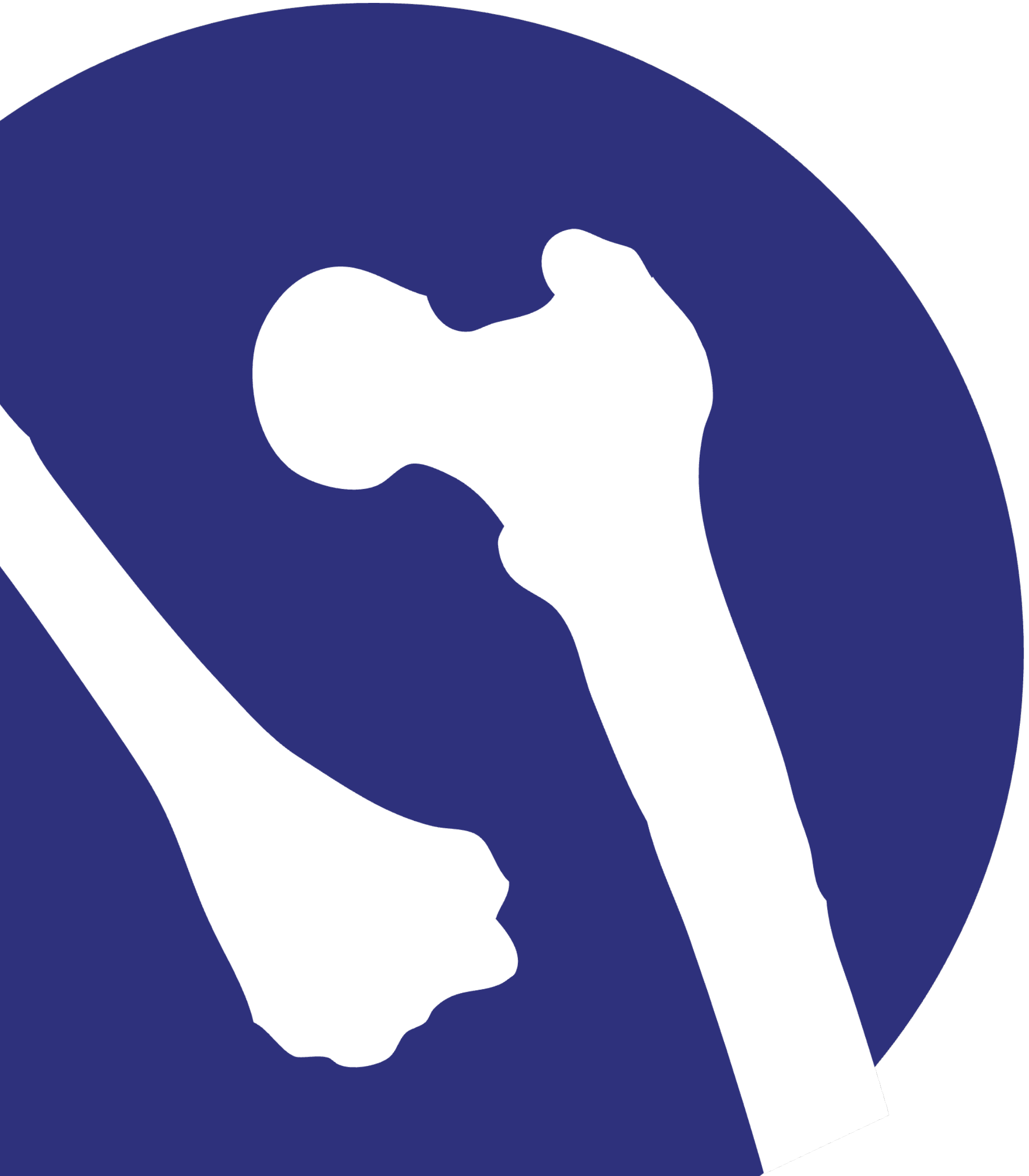

Product Introduction

What is the Orth-Bridge System?

OBS.

LEPU MEDICAL

OBS = Rods + Clamps + Screws

Standard Clamps

Clamp 02
Single-Rod-Single-Hole
Φ3、Φ4、Φ5、Φ6

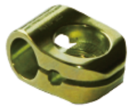

Clamp 02
Single-Rod-Single-Hole
(Half Hook)
Φ3、Φ4、Φ5、Φ6

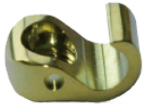

Clamp 04
Single-Rod-Double-Hole
Φ3、Φ4、Φ5、Φ6

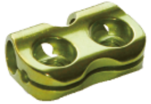

Clamp 04
Single-Rod-Double-Hole
(Half Hook)
Φ3、Φ4、Φ5、Φ6

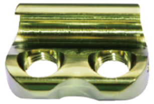

Clamp 16
Double-Rod-Single-Hole
Φ3、Φ4、Φ5、Φ6

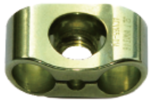

Clamp 16
Double-Rod-Single-Hole
Φ4-Φ5

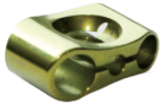

Clamp 07
Double-Rod-Double-Hole
Φ3、Φ4、Φ5、Φ6

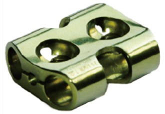

Clamp 07
Double-Rod-Double-Hole
Φ4-Φ5

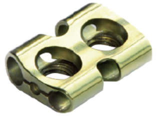

Clamp 01
Φ3、Φ4、Φ5、Φ6、
Φ4-Φ5、Φ5-Φ4

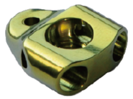

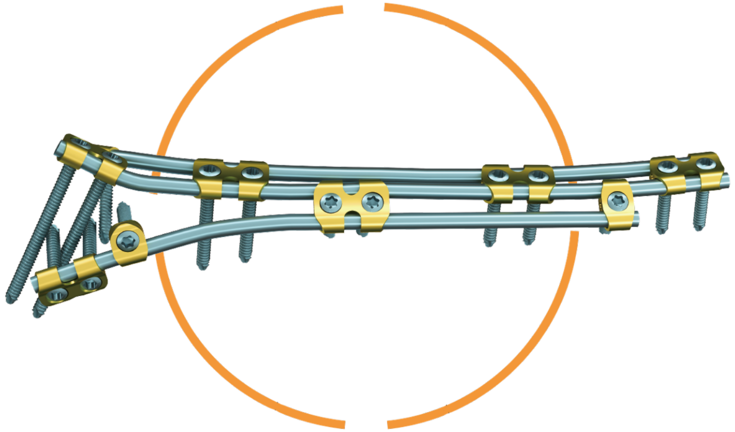

Anatomical Clamps

Clamp 12
Proximal Femoral Anatomical
Clamp
Φ6(L/R)

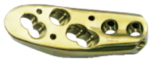

Clamp 17
Distal Femoral Anatomical
Clamp
Φ6(L/R)

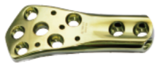

Clamp 15
Proximal Lateral Tibial
Anatomical Clamp
Φ4.0-Φ5.0(L/R)

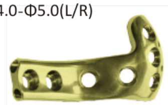

Clamp 14
Proximal Medial Tibial
Anatomical Clamp
Φ4.0-Φ5.0(L/R)

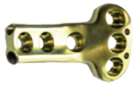

Clamp 23
Distal Lateral Tibial
Anatomical Clamp
Φ4.0-Φ5.0(L/R)

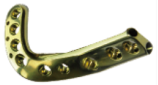

Clamp 10
Proximal Humeral Anatomical
Clamp
Φ4.0

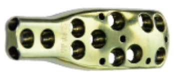

Set Screw
M4.5/M6.0

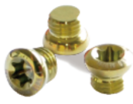

Screw
Φ4.0/Φ5.0

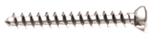

Locking Screw
Φ3.5/Φ4.5/5.0

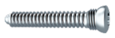

Connecting Rod 05
Φ3、Φ4、Φ5、Φ6

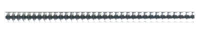

Connecting Rod 01
Φ3、Φ4、Φ5、Φ6

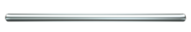

OBS

LEPU MEDICAL

OBS = Rods + Clamps + Screws

Anatomical Clamps

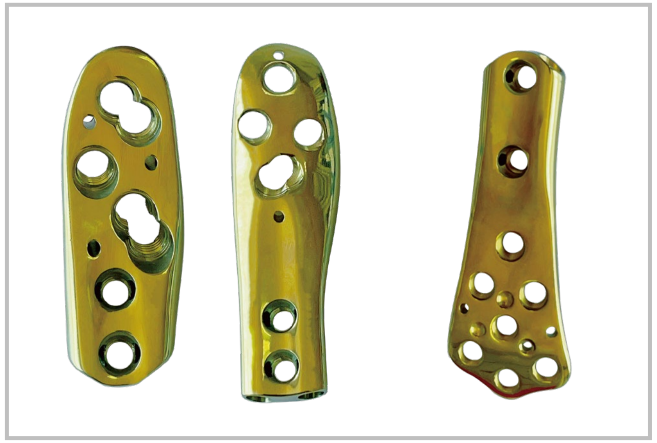

Proximal Femur

Distal Femur

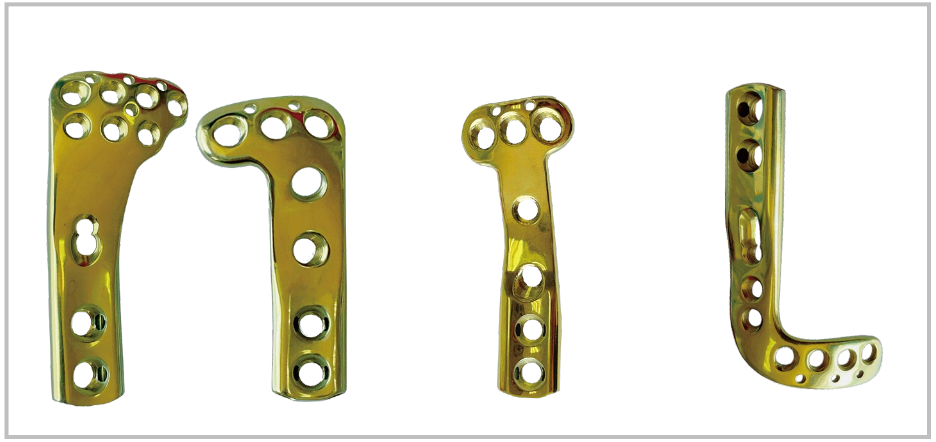

Proximal Lateral Tibia

Proximal Medial Tibia

Distal Lateral Tibia

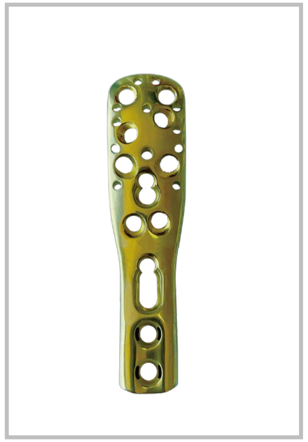

Proximal Humerus

OBS

LEPU MEDICAL

OBS = Rods + Clamps + Screws

Standard Clamps

Clamp 02
Single-Rod-Single-Hole
Φ3、Φ4、Φ5、Φ6

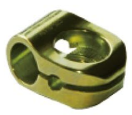

Clamp 02
Single-Rod-Single-Hole
(Half Hook)
Φ3、Φ4、Φ5、Φ6

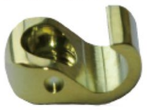

Clamp 04
Single-Rod-Double-Hole
Φ3、Φ4、Φ5、Φ6

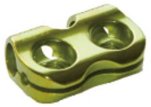

Clamp 04
Single-Rod-Double-Hole
(Half Hook)
Φ3、Φ4、Φ5、Φ6

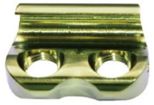

Clamp 16
Double-Rod-Single-Hole
Φ3、Φ4、Φ5、Φ6

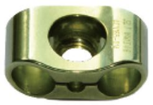

Clamp 16
Double-Rod-Single-Hole
Φ4-Φ5

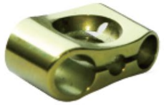

Clamp 07
Double-Rod-Double-Hole
Φ3、Φ4、Φ5、Φ6

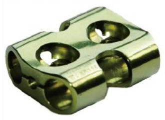

Clamp 07
Double-Rod-Double-Hole
Φ4-Φ5

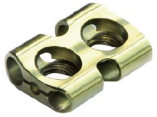

Clamp 01
Φ3、Φ4、Φ5、Φ6、
Φ4-Φ5、Φ5-Φ4

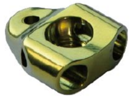

PRINCIPLE

Rods: for connecting and shaping

Clamps: for structuring, and for positioning the screws

Screws: for fixation of the fractures and the system

Anatomical Clamps

Clamp 12
Proximal Femoral Anatomical Clamp
Φ6(L/R)

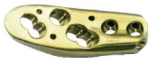

Clamp 17
Distal Femoral Anatomical Clamp
Φ6(L/R)

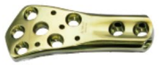

Clamp 15
Proximal Lateral Tibial Anatomical Clamp
Φ4.0-Φ5.0(L/R)

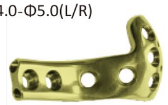

Clamp 14
Proximal Medial Tibial Anatomical Clamp
Φ4.0-Φ5.0(L/R)

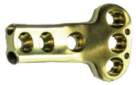

Clamp 23
Distal Lateral Tibial Anatomical Clamp
Φ4.0-Φ5.0(L/R)

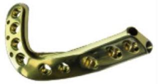

Clamp 10
Proximal Humeral Anatomical Clamp
Φ4.0

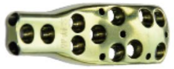

Set Screw
M4.5/M6.0

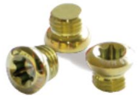

Screw
Φ4.0/Φ5.0

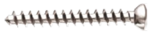

Locking Screw
Φ3.5/Φ4.5/5.0

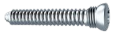

Connecting Rod 05
Φ3、Φ4、Φ5、Φ6

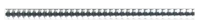

Connecting Rod 01
Φ3、Φ4、Φ5、Φ6

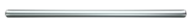

OBS

LEPU MEDICAL

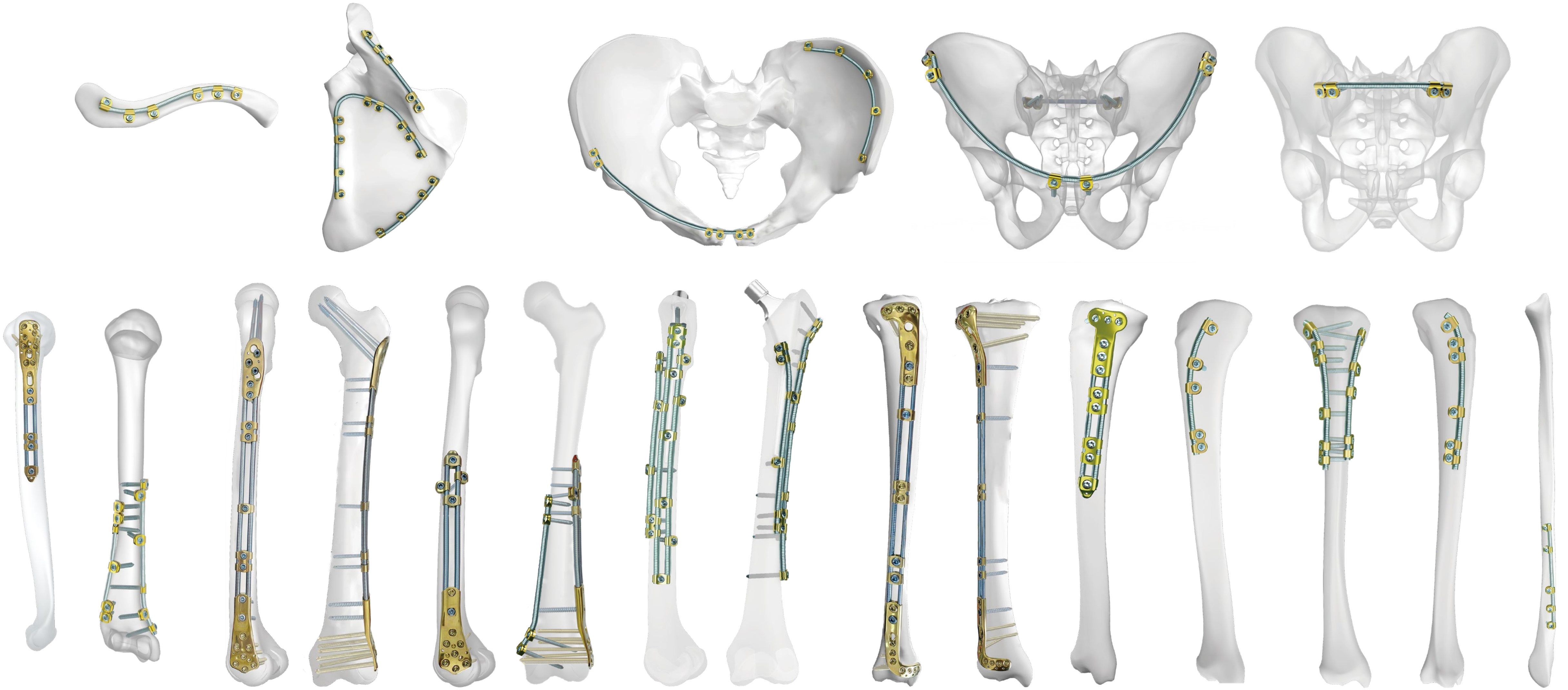

Supplement: Supplementary File S1 — The Ortho-Bridge System introduction which come from the official website of Walkman Biomaterial Co., Ltd. [file Datasheet1.pdf]
